# Recent advances in understanding pancreatic cancer

**DOI:** 10.12703/r/11-9

**Published:** 2022-04-20

**Authors:** Martyn C Stott, Lucy Oldfield, Jessica Hale, Eithne Costello, Christopher M Halloran

**Affiliations:** 1 Department of Molecular & Clinical Cancer Medicine, Institute of Systems, Molecular and Integrative Biology, University of Liverpool, Sherrington Building, Liverpool, UK

**Keywords:** Pancreatic ductal adenocarcinoma (PDAC), early detection, high-risk groups, artificial intelligence, tumour stroma, immune microenvironment, tumour microbiome

## Abstract

Pancreatic ductal adenocarcinoma (PDAC) is an intractable cancer and a leading cause of cancer deaths worldwide. Over 90% of patients die within 1 year of diagnosis. Deaths from PDAC are increasing and it remains a cancer of substantial unmet need. A number of factors contribute to its poor prognosis: namely, late presentation, early metastases and limited systemic therapy options because of chemoresistance. A variety of research approaches underway are aimed at improving patient survival. Here, we review high-risk groups and efforts for early detection. We examine recent developments in the understanding of complex molecular and metabolic alterations which accompany PDAC. We explore artificial intelligence and biological targets for therapy and examine the role of tumour stroma and the immune microenvironment. We also review recent developments with respect to the PDAC microbiome. It is hoped that current research efforts will translate into earlier diagnosis, improvements in treatment and better outcomes for patients.

## Introduction

**Figure 1.**
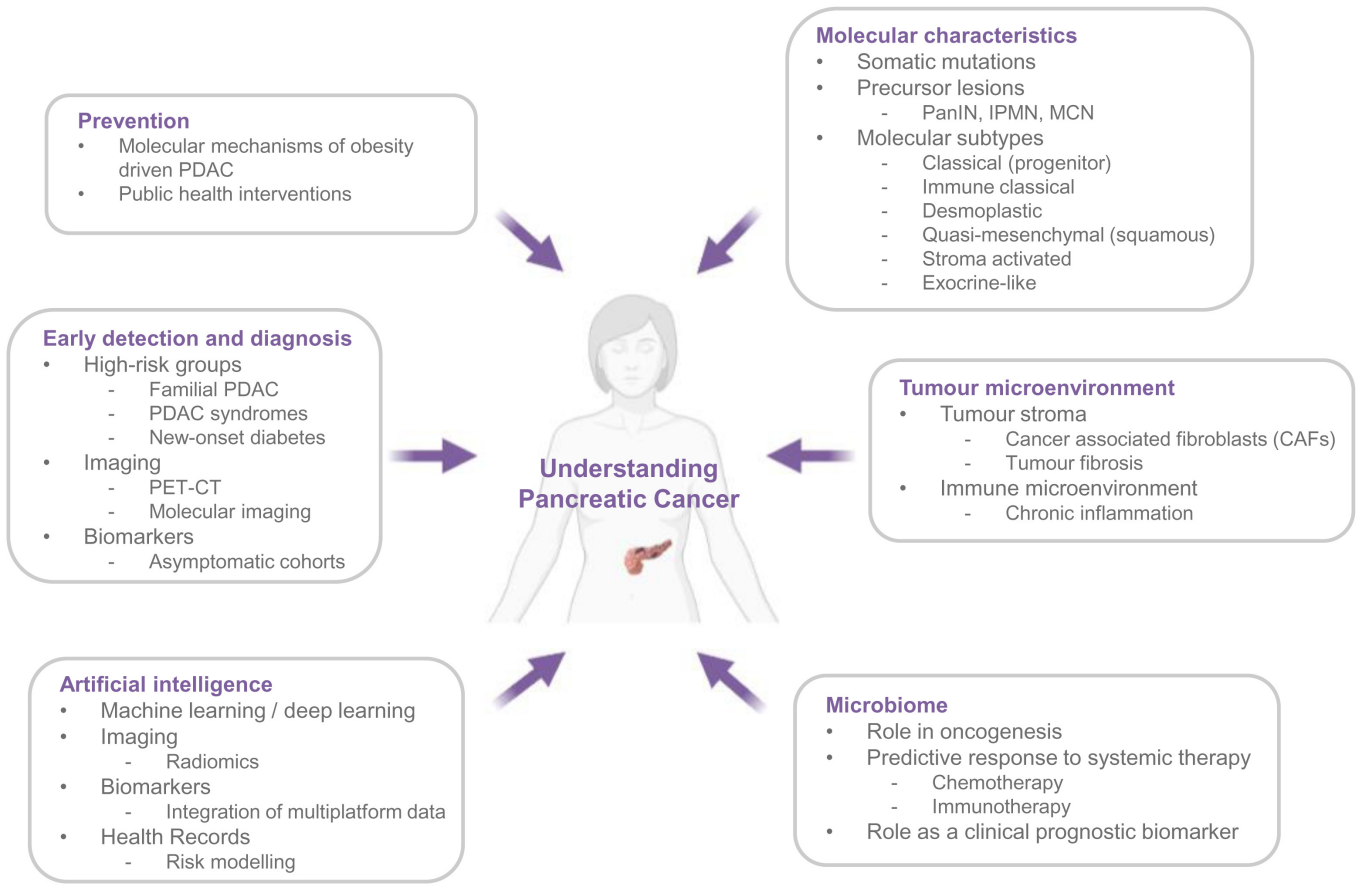
Research areas contributing to our understanding of pancreatic cancer. IPMN, intraductal papillary mucinous neoplasm; MCN, mucinous cystic neoplasm; PanIN, pancreatic intraepithelial neoplasia; PDAC, pancreatic ductal adenocarcinoma; PET-CT, positron emission tomography–computed tomography.

Pancreatic ductal adenocarcinoma (PDAC) has a 5-year survival of around 7 to 10%, which remains almost unchanged in 40 years^1,2^. Despite advances in uncovering the biology underpinning this disease, and improvements in diagnostic and cancer registration practices in some countries, deaths from PDAC are projected to increase in the coming years^2^. The only possibility of cure is appropriate surgery with adjuvant chemotherapy^3^. Patients classically present with obstructive jaundice (when the tumour arises in the pancreatic head); however, many present with vague symptoms if tumours arise in the body or tail of the pancreas^4^. Clinical vigilance is key. Thus, at presentation, almost 80 to 85% already have locally advanced or distant metastatic disease, and treatment options offering curative intent are limited^5,6^. Although patients can be brought to surgery using neo-adjuvant regimens^7^, those with metastatic disease are currently unsuitable for surgery. The success of systemic therapies has also been limited because of the intense resistance of the disease to current treatment regimens. Progress in treatment, including personalised approaches, in combination with earlier detection could significantly contribute to better patient outcome (Figure 1).

## PDAC prevention

Risk factors for PDAC include a history of familial pancreatic cancer (FPC)^8^, hereditary syndromes predisposing to PDAC^9^, intraductal papillary mucinous neoplasms (IPMNs) and mucinous cystic neoplasms^10^, chronic pancreatitis^11^, (especially when related to inherited homologues of human cationic trypsinogen [*PRSS1*]), obesity^12^, new-onset diabetes (NOD)^13^ and smoking^14^. The European Prospective Investigation into Cancer and Nutrition (EPIC) highlighted that individuals with high healthy lifestyle index scores (including measures of smoking, alcohol consumption, physical activity, adiposity and diet) have a decreased likelihood of developing PDAC^15^.

The rise in obesity and diseases associated with it, including type 2 diabetes, remains a major significant public health challenge in higher socio-economic index countries and increasingly in the developing world. Evidence from case-control and cohort studies suggests an association between obesity and PDAC^16–18^. Similarly, some animal and small case-control studies in humans have related fatty infiltration of the pancreas to PDAC development^19^. It has been suggested that obesity may drive tumorigenesis through the activation of KRAS signalling pathways, although our understanding of this process is in its infancy^20^. Understanding the molecular mechanisms behind obesity-driven tumorigenesis would facilitate the development of risk reduction strategies and targeted screening for obese individuals. Furthermore, understanding how to effectively implement public health interventions will be key in primary prevention at a population level.

## Early detection and diagnosis of PDAC

Worldwide, there are currently no recommended population-wide screening programmes for PDAC in asymptomatic adults and this is due in part to the relatively low incidence of the disease in the general population (1.5–10 per 100,000 age-standardised rate, worldwide^2^). The World Health Organization advocates screening in all cancers, and anticipated increases in curative treatment are up to 30% from earlier detection (www.who.int/cancer/en/index.html). Five per cent to 10% of cases of PDAC are due to inherited risk factors: either a family history of the disease or germline mutations which give rise to PDAC syndromes^9^. The most studied of those syndromes include Peutz-Jeghers syndrome (*STK11*)^21^, hereditary breast-ovarian syndrome (*BRCA2*)^22^, familial atypical multiple mole melanoma (*CDKN2A*)^23^ and Lynch syndrome (*MLH1/2/6*)^24^. The International Cancer of the Pancreas Screening (CAPS) Consortium recommends screening for high-risk individuals, notably carriers of high-risk germline mutations, such as those mentioned above, or individuals with defined FPC. This screening should be offered in a research setting until the benefits, risks and costs of pancreatic cancer surveillance are elucidated^25^. Established research screening programmes include the following: the European Registry of Hereditary Pancreatitis and Familial Pancreatic Cancer (EUROPAC), Liverpool, UK^26^; the North American National Familial Pancreatic Tumour Registry^27^ and the German National Case Collection for FPC^28^. There are specific guidelines for screening patients with chronic pancreatitis for the risk of pancreatic cancer. The current literature supports screening only for those patients with an autosomal dominant history of hereditary pancreatitis with or without a mutation in cationic trypsinogen (*PRSS1* gene)^29^.

Individuals with NOD are the largest high-risk group who may benefit from screening^30^. The relationship between diabetes and PDAC is bidirectional^31^, and long-standing diabetes has a pooled relative risk of 2.1 for PDAC development, while individuals with NOD (duration of less than 1 year) have a 5.4-fold relative risk of developing PDAC^32^. For about 1% of individuals who are at least 50 years old and whose diabetes has been diagnosed, PDAC is the underlying cause of diabetes. PDAC-associated diabetes is a form of type 3c diabetes^33^. Hyperglycaemia in PDAC occurs up to 3 years before diagnosis and begins before pancreatic tumours are visible on imaging, suggesting that the pathophysiology of PDAC-associated glucose dysregulation is more than the destruction of the gland^34^. Further support for this is shown by the improvement in glucose dysregulation after surgical resection of the tumour^35^. Despite recent advances, our understanding of the biology underpinning the development of diabetes in PDAC is in its infancy. However, with about 80% of individuals presenting with glucose dysregulation at the time of diagnosis of PDAC, including a large proportion presenting with NOD, screening strategies for this high-risk group are urgently needed^30^. Cohorts of patients in both the USA (Chronic Pancreatitis, Diabetes and Pancreatic Cancer Consortium^36^) and the UK (UK Early Detection Initiative for Pancreatic Cancer^37^) have been established in response to this need to investigate how the presence of NOD may be used to expedite the detection of PDAC in this high-risk group.

The capacity for early detection is limited by current imaging modalities, which lack the sensitivity to identify small lesions. Endoscopic ultrasound (EUS), which has a sensitivity of 72% for identifying T1 and T2 cancers, is advocated in FPC but suffers from inter-user variability, inconsistency in patient access, and invasiveness^38^. Cross-sectional imaging is used for screening patients with hereditary pancreatitis, as the calcium load in these individuals will prevent adequate EUS assessment. Future developments with positron emission tomography (PET) and the use of molecular imaging techniques that target proteins overexpressed by the tumour, signalling pathways, or the stroma may improve detection of early lesions^39,40^. Further development of hyperpolarised magnetic resonance imaging, which can identify metabolic aberrations in the pancreas indicative of pre-neoplasia, may also prove to be a useful adjunct to early detection and screening^41^.

It is widely accepted that biomarkers will be essential in the refining of inclusion criteria for future PDAC screening programmes. Much research has been published in this area; however, no biomarkers have been carried through to clinical use. The failure to translate biomarker development into the clinic lies in part with the widespread validation of biomarkers using a diagnostic sample, which are compromised by late changes during tumorigenesis that are not seen in early-stage disease. Second, where existing pre-diagnostic cohorts exist, they often lack adequate control groups and do not contain data relevant to PDAC, such as diabetes status or presence of chronic pancreatitis, limiting the interpretation of findings. Finally, and perhaps most importantly, PDAC within the population is relatively rare, which demands high levels of specificity from biomarkers to minimise false-positive rates. To comprehensively test biomarker accuracy, large numbers of samples are required from target populations and these cohorts are hard to come by. Of those published biomarkers, carbohydrate antigen (CA) 19-9 remains the only validated biomarker clinically used in the management of PDAC. Although CA19-9 may hold future utility as a component in a panel of markers for the identification of PDAC, it lacks the sensitivity and specificity to act as a stand-alone biomarker for asymptomatic disease^42–44^. As our understanding of the molecular changes that occur during the transition from pre-neoplastic lesion to early PDAC and advanced disease improves, rational strategies have been applied to identify novel biomarkers across genomic, transcriptomic, metabolomic and proteomic platforms utilising a variety of specimens, including microvesicles (exosomes), ctDNA and methylated DNA^45–47^. New pre-diagnostic cohorts and large collaborative studies will be crucial to the development of biomarkers and their rapid translation to the clinic. 

To meet with the demand for rapid evaluation of an increasing number of early-stage tumours, it is likely that diagnostic, prognostic and predictive algorithms, based on a combination of imaging, molecular and health data, will be needed. Here, artificial intelligence (AI), and more specifically machine learning (ML) and deep learning (DL) (a subset of ML applied to large datasets), have emerged as valuable tools for disease risk-stratification and identification in general populations. ML and DL automate analytical model building. They use a variety of operations to examine and compare small to large datasets to find common patterns and explore nuances. Both ML and DL can be performed in a supervised or unsupervised manner, with unsupervised learning training models and unlabelled data.

Early detection and management of pancreatic cancer rely on an understanding of the basic biology of the disease as well as an appreciation of the disease course at a population level (risk factors, disease progression, and treatment response). AI methods are making contributions here across the spectrum of basic research areas, and progress requires interdisciplinary collaboration between experts in AI, disease physiology and molecular cell biology. Perhaps most widely explored is the use of ML and DL approaches for the extraction and interpretation of features in medical imaging, although we are still only just scratching the surface of what is possible for image-based modelling of pancreatic cancer risk^48^. More recently, AI methods (ML and DL) have been explored to provide a better quantitative description of biological processes in PDAC^49^, to identify novel biomarkers^50^, and to undertake large-scale analysis of clinical records to develop algorithms for automatic identification of at-risk individuals^51^.

Increasingly extensive datasets are being generated as a result of advances in high-throughput proteomic, transcriptomic and genomic technologies. As ML researchers and practitioners gain more experience, the capacity for ML to interrogate and integrate this information to aid in our understanding of PDAC biology, and to inform how to manage and detect the disease at the earliest time point, will be immense. This will be an exciting area to follow in the coming years.

## Targeting the molecular characteristics of PDAC

PDAC is preceded by the progression of precursor lesions, such as pancreatic intraepithelial neoplasia (PanIN), IPMN and mucinous cystic neoplasm. PanIN, which arises from a stepwise accumulation of somatic genetic mutations, is the most common of these premalignant lesions and represents a key target for early detection and treatment^52^. The major genetic mutations responsible for PanIN progression are well established. Grade 1 and 2 PanIN are characterised by a point mutation in the *KRAS* oncogene (90% of tumours)^53^, and telomerase shortening is also characteristic of this stage^54^. Grade 2 is associated with increased activation of *CDKN2A* and *CDKN1A*^55^. Grade 3 and 4 are associated with mutations in *TP53* (50–70%) and *SMAD4* (60–90%)^56,57^. There are many more somatic mutations described in PDAC specimens, and over 100 to 150 have been identified using next-generation sequencing^58^. Most somatic mutations occur in common pathways: RAS signalling, transforming growth factor beta (TGFβ) pathways, cell-cycle control, WNT signalling, NOTCH signalling and DNA damage repair^59^. As in other gastrointestinal tumours, the majority of somatic mutations are not currently targetable; however, there is some evidence that our understanding of underlying mutations is translating into clinical practice, including small molecule inhibitors of KRAS^G12C^ and PD-1 blockade in tumours deficient in the mismatch repair (MMR) genes *MLH1* and *MLH2*^60,61^*.*

Recent global transcriptomic analysis has allowed PDAC to be subclassified based on distinct molecular signatures. Early work classified these molecular subtypes as classical (progenitor), quasi-mesenchymal (squamous) and exocrine-like (aberrantly differentiated endocrine exocrine), although this classification was based on analysis of the epithelial aspects of PDAC tumours only and did not take into account the wider tumour microenvironment (TME)^62^. A more recent analysis of 309 resected PDAC tumours confirmed the progenitor, squamous and quasi-mesenchymal subsets but also further characterised the TME, identifying three additional subsets: desmoplastic, immune classical and stroma-activated^63^. It has been suggested that tumours of the squamous subset are associated with an adverse prognosis and are more resistant to current chemotherapy regimens compared with other subtypes; however, fully translating this work into clinical applications is still in the early stages^64^.

There are a number of research programmes aiming to apply the expansion of molecular subtypes of PDAC into clinical trials, and this is a major area of collaboration across industry and academia. The “Know Your Tumour Programme”, one such example, aims to determine whether targeting actionable molecular signatures can improve outcomes^65,66^. Much of this programme is focussed on patients with metastatic disease, and the most common actionable alterations are seen in DNA repair genes (*BRCA1/2* or *ATM* mutations) or cell-cycle genes (*CDK4/6* alterations). It should be noted that, despite genetic testing, the majority of patients (68%) enrolled in this trial nonetheless underwent standard-of-care chemotherapy with either FOLFIRINOX or gemcitabine with nab-paclitaxel rather than targeted therapy^66^. Other classes of drugs utilised based on molecular signatures of PDAC include inhibitors of MEK^67^ and PARP^68^. Data published from the “Know Your Tumour Programme” suggest that patients who underwent therapy that were matched to molecular profiling had better overall survival than those who did not (2.58 versus 1.51 years; *P* = 0.004), suggesting that it is feasible to tailor therapy on the basis of individual molecular characteristics. However, of the 1,856 patients who were referred to the programme, only 46 (2%) underwent matched therapy and this was due to the aggressiveness of the disease and limited treatments available at the time of the trial for some of the identified molecular signatures, such as *NTRK* alterations^65,69^. Additional limitations relating to targeted therapies should be noted. These include the difficulty in obtaining samples of adequate yield and quality for molecular subtyping in non-resected patients following fine-needle aspiration as well as intra-tumour heterogeneity of PDAC^70^. Targeted treatments based on molecular profiling, therefore, can currently be viewed as an encouraging proof-of-concept idea that requires ongoing global efforts before the majority of patients will benefit from such methods.

## The role of tumour stroma in resistance to treatment

PDAC exhibits a strong desmoplastic reaction characterised by a hypoxic and hypovascular TME. This desmoplasia is argued to be a principal contributor of resistance to standard chemotherapy in PDAC^71^. Tissue stroma plays a role in the response to injury utilising the immune, vascular and connective tissue components within it. Understanding and trying to develop treatments to target aspects of the TME have therefore been the focus of research over the last decade. The characteristic dense stroma induced by PDAC includes an array of cell types, including cancer-associated fibroblasts (CAFs), inflammatory cells, blood vessels and nerve cells, as well as the extracellular matrix (ECM) produced by the CAFs, including collagen, fibronectin, laminin and hyaluronic acid^72,73^.

Pancreatic stellate cells (PSCs) are myofibroblast-like cells that are activated by PDAC cells and become CAFs, producing ECM resulting in fibrosis in the tumour^74^. This resultant desmoplasia creates a mechanical barrier around tumour cells and prevents vascularisation, which limits the delivery of chemotherapy and also immune cell infiltration^75^. Thus, a key area of research is to understand how to target the development of this dense stroma, either by targeting the extracellular components of the fibrosis itself or by targeting stromal cells (PSCs) and attempting to revert them to their quiescent form. Matrix metalloproteinases (MMPs) are a group of proteins that remodel the ECM. MMP2^76^, MMP7^77,78^ and MMP9 and tissue inhibitors of MMPs^79^ are shown in pre-clinical models to be differentially expressed between normal pancreas and PDAC, and higher levels are associated with worse prognosis and metastatic disease^80^. Marimastat^81^ and tanomastat^82^, two inhibitors of MMP, showed promising results in pre-clinical xenograft models of melanoma^83^, gastric cancer^84^ and colon cancer^85^. When these were applied to metastatic PDAC in phase 3 trials, however, there was no survival benefit^81,82^.

Interest in targeting hyaluronan within the ECM, stemming from the observation that high deposition of hyaluronan in PDAC is associated with poor prognosis, has developed over a number of years^86^. PEGPH20 is a human recombinant PH20 hyaluronidase, which in a mouse model led to depletion of hyaluronan, improved vascular permeability and increased drug delivery of gemcitabine and chemotherapeutic efficacy^86^. Clinical trials, however, have shown differing results; phase II trials of PEGPH20 with gemcitabine plus nab-paclitaxel have improved progression-free survival but reduced overall survival when combined with FOLFIRINOX^87,88^. Also, a subsequent phase III trial combining PEGPH20 with gemcitabine plus nab-paclitaxel did not improve overall survival compared with chemotherapy alone (hazard ratio = 1.00, *P* = 0.97)^89^. Much of this was due to the side effects of PEGPH20 plus the worsening of chemotoxic symptoms with FOLFIRINOX leading to reduced treatment regimens and dosages.

Subsequently, attempts to target signalling pathways responsible for the development of stroma rather than particular components of the ECM have focussed on the hedgehog signalling pathway. *In utero*, repression of endodermal Sonic hedgehog (SHH) by inhibin-βB and FGF2 allows the expression of *Pdx1* and insulin, initiating pancreatic differentiation^90^. Dysregulated hedgehog signalling is implicated in pancreatic carcinogenesis^91^, and mouse models showed that hedgehog signalling promotes desmoplasia and antibody-mediated inhibition reduced this desmoplastic reaction^92^. There is also some evidence of paracrine stimulation of PSCs through this pathway^93^. Translation of this in phase II studies, however, has been disappointing. A trial combining the SHH inhibitor saridegib and gemcitabine in metastatic PDAC was stopped early in 2012 because it led to higher rates of progressive disease^94^. Similar trials combining gemcitabine with vismodegib also showed no benefit to overall or disease-free survival compared with chemotherapy alone^95^.

Efforts to target CAFs and PSCs in an attempt to reduce the desmoplastic reaction have focussed mainly on inhibition of fibroblast activation protein (FAP)^96^. Similar to results in colorectal cancer, the success of such treatments in PDAC has been limited^96^. The use of a small molecule inhibitor UAMC-1110 was not effective in a recent mouse model^97^. Indeed, studies of genetic deletion of fibroblasts in mouse models of PanIN and PDAC led to disease with more aggressive phenotypes, indicating that fibroblasts play a complex role in tumour development^98^. CAFs are also shown to be important for shaping the antitumour immune response^99^. Of particular importance is the role of CXCL12-CXCR4 signalling in stromal-immune crosstalk^100^. The COMBAT trial combined motixafortide, a CXCR4 inhibitor, with PD-1 inhibition in a phase IIa open-label study in metastatic PDAC and showed modest but insignificant changes to overall survival^101^. Thus, it is likely that future efforts will be focussed on exploiting reprogramming of the fibroblasts and the role they play in altering the TME rather than the cruder method of attempting to eliminate them. The results of ongoing studies focussing on CXCR4 inhibition (cemiplimab; ClinicalTrials.gov Identifier: NCT04177810) are eagerly awaited.

## Understanding the immune microenvironment

The TME in PDAC is rich with immune cells, and it has long been established that chronic inflammation is an important characteristic of PDAC^102^. However, PDAC is a relatively immunologically “cold” tumour, and molecular profiling indicates that only a subset of the immune cells present within the tumour are immunologically active^64,103^. With respect to immune cells, the myeloid compartment dominates the TME and includes tumour-associated macrophages (TAMs), granulocytes and inflammatory monocytes. These are actively recruited to the TME during carcinogenesis orchestrated by *KRAS* mutations in the epithelial compartment^104^. TAMs can be categorised as either M1 or M2 macrophages; M1 generally shows tumoricidal activity acting via tumour necrosis factor (TNF), interleukin-12 (IL-12), IL-1α and interferon-gamma (INF-γ), and M2 produces anti-inflammatory tumour-promoting cytokines such as TGFβ and IL-10^105^. Myeloid-derived suppressor cells (MDSCs) are also produced from myeloid cells and these recruit regulatory T cells to the TME^106,107^. There is also evidence to suggest that this myeloid cell infiltration is critical for PDAC initiation (induction of immune checkpoint ligands) and it promotes the formation and maintenance of pre-neoplastic lesions^108^.

Immunotherapy is an active area of interest, most commonly aimed at augmenting the antitumour adaptive immune response. Most of the above-mentioned immunosuppressive cells, including TAM M2 macrophages^109^, TAM M1 macrophages^110^ and MDSCs^111^, have been targeted. Further targets include CD40 agonists^112,113^, chemokine modulation^114^ and immune checkpoint inhibitors^115^. Despite showing promise in other solid organ tumours, the most studied of these—CTLA-4 (ipilimumab), and PD-1 (nivolumab)—have been disappointing in PDAC^116^. Another approach is to enhance antigen presentation and drive the expansion of tumour-specific T-cell clones through “vaccination”. Whilst multiple studies have shown that it is possible to yield antigen-specific immunological responses in patients with PDAC, vaccination strategies alone might not be enough to generate clinically meaningful antitumour effects^117^.

The limitations of direct stroma or immune-based therapeutic targeting perhaps allude to the logic that a better strategy would be to exploit integrated aspects of the TME, such as specific points of biological convergence. An example of this is targeting cancer cell metabolism. Cancer cells maintain high glycolytic activity in order to grow as well as needing glutamine to fuel the tricarboxylic acid cycle (TCA) cycle^118,119^. It has been suggested that there is a symbiotic relationship between PDAC cells and the microenvironment, including CAFs and TAMs. CAFs release non-essential amino acids through enhanced autophagy to support tumour cell needs through the TCA cycle^120^. Moreover, there is some evidence that PDAC cells reprogramme the stroma into a tumour-promoting metabolic environment that hinders T cells^121^. Blocking glutamine metabolism augmented with anti-PD-1 led to cytotoxic T-cell activation and a reduction in hyaluronan synthesis in a mouse model of PDAC^122,123^. There is much to learn in this area, and limited data are available from the small number of trials conducted. However, these studies remain of great interest as they suggest that focussing on the metabolic remodelling of the TME may influence desmoplasia, cancer metabolism and the immune response in a more orchestrated way.

## The role of the pancreas microbiome

Characterising the pancreatic tumour microbiome is providing insight into carcinogenesis. It is also uncovering the potential of the tumour microbiome as a therapeutic biomarker. Whilst the presence of bacteria in tumours is well recognised, their exact purpose or the consequences of their presence remain unclear. In an attempt to answer this question, Nejman *et al*. profiled the bacteria present in seven different human tumours, demonstrating that bacteria were located intracellularly in both cancer and immune cells^124^, raising the possibility that they influence the immune state of the tumour environment and have potential implications for responses to immunotherapy. Interestingly, the bacterial composition of tumours varied between tumour types.

The notion that certain microbes play a causative role in oncogenesis is gaining momentum^125,126^. Significant variation in methodology and results, however, currently prevents consensus opinion. Elevated levels of intracystic bacterial DNA were found in patients with IPMN (both with high-grade dysplasia and with cancer)^127^, raising pertinent questions regarding the potential of iatrogenic bacterial translocation via endoscopy. Of course, it must be appreciated that other routes of translocation exist. Furthermore, exploration of pancreatic cystic fluid (PCF) has revealed the existence of a unique bacterial ecosystem, which may play a role in oncogenesis^128^.

The pancreatic cancer microbiome may also act as a clinical prognostic biomarker. Via 16S rRNA gene sequencing, the tumour microbiome of long-term survivors (LTSs) was compared with that of short-term survivors (STSs)^129^. LTSs were found to have a more diverse tumour microbiome, and an intra-tumoral microbiome signature was found to be predictive of long-term survival. Human-to-mice faecal microbial transplants demonstrated attenuated tumour growth and immune cell infiltration with LTS faeces when compared with STS faeces. This pivotal study demonstrated that crosstalk occurs between the gut and tumour microbiome, which can directly influence and predict the outcome of disease^129^.

The immunosuppressive environment of PDAC to date has hindered the effective use of immunotherapy. Targeting the intra-tumoural microbiome may be a strategy to increase efficacy. Bacterial ablation of tumours results in immunogenic reprogramming of the PDAC microenvironment, reducing MDSCs, increasing macrophage differentiation, and promoting CD4^+^ T helper cells and CD8^+^ T-cell activation^130^. Reducing the bacterial content of tumours upregulates PD-1 expression, increasing the efficacy of checkpoint-targeted immunotherapy^130^. A number of studies in tumours such as lung and melanoma have addressed the proposal that gut dysbiosis may affect response to treatments^131–133^. The idea that we may be able to use microbiota composition to define groups most likely to respond to treatment is gaining traction and may help inform future clinical trials.

It can no longer be assumed that the bacterial populations found inside and around PDAC are merely environmental bystanders. What remains unclear is whether bacteria in these regions contribute to causing the cancer or whether they populate as a result of oncogenesis. What is recognised, however, is that crosstalk between organs and their microbiomes exists and is far greater than anticipated. A barrier to translating scientific findings into large-scale clinical trials and clinically meaningful results is the issue of bias in the studies published, which can occur at any stage of the analysis. Consequently, there are relatively few clinical trials aimed at evaluating therapies manipulating the pancreatic microbiome^134^. The results from emerging clinical trials in this area are thus eagerly awaited.

## Conclusions

PDAC remains a major global health problem. Improvements in survival have been made over the last 20 years because of advances in perioperative care, meaning that patients who undergo surgery can also benefit from adjuvant therapy. Many patients continue to present late with a disease that is too advanced for surgical intervention with curative intent, and this remains a significant challenge. Our understanding of high-risk groups and how to detect disease early in these groups continues to be central to improving outcomes, and ongoing research using high-risk cohorts is vitally important. Developments in AI and ML will hopefully improve early detection initiatives taking into account large datasets. There has been an increase in our understanding of the biology of PDAC, its microenvironment and the microbiome across the genetic, epigenetic, transcriptomic, proteomic and metabolomic spectrums. The translation of these findings into clinical trials remains an aim for the future.
